# Pulmonary hemorrhage syndrome associated with dengue fever, High-resolution computed tomography findings: a case report

**DOI:** 10.1186/1750-1172-4-8

**Published:** 2009-03-05

**Authors:** Edson Marchiori, José Luiz N Ferreira, Carolina N Bittencourt, César A de Araújo Neto, Gláucia Zanetti, Cláudia M Mano, Alair ASD Santos, Alberto D Vianna

**Affiliations:** 1 Fluminense Federal University, Rua Thomaz Cameron, 438, Valparaiso, CEP 25685.120, Petrópolis, Rio de Janeiro, Brazil; 2 Hospital São Rafael, Salvador, Rua Dr Antonio Monteiro 105, Salvador, Bahia, Brazil; 3 Hospital Otavio Mangabeira, Rua Pacifico Pereira, 457, Garcia, Salvador, Bahia, Brazil; 4 Bahia Federal University, Rua Professor Clementino Fraga, 198, Ondina, Salvador, Bahia, Brazil; 5 Petrópolis Faculty of Medicine, Rua Coronel Veiga, 733, Centro, Petrópolis, Rio de Janeiro, Brazil; 6 Fluminense Federal University, Rua Dr Paulo Alves, 110, Ingá, Niterói, Rio de Janeiro, Brazil

## Abstract

Dengue hemorrhagic fever is an acute infectious disease caused by dengue virus. We described the high-resolution CT findings in a 70-year-old male with the disease, which was diagnosed by clinical examination and confirmed by serological methods. High-resolution CT demonstrated bilateral areas of consolidation with air bronchogram and ground glass opacities, as well as small bilateral pleural effusions. Dengue hemorrhagic fever should be considered in the differential diagnosis of diffuse pulmonary hemorrhage.

## Background

Dengue fever (DF) is an acute infectious disease caused by dengue virus (DENV). It is a mosquito-borne flavivirus that belongs to the family *Flaviviridae*, and consists of four distinct serotypes (DENV 1–4) [1,2]. Dengue virus causes disease in humans, including dengue fever, dengue hemorrhagic fever (DHF) and dengue shock syndrome (DSS) [1,2]. The virus is transmitted to humans by the bite of infected female mosquitoes of the genus *Aedes*. Dengue disease has a wide spectrum of clinical signs and symptoms, ranging from asymptomatic infection to severe and lethal manifestations [3]. However, pulmonary hemorrhage and hemoptysis have been rarely described in the literature [4,5].

The prevalence of dengue infection has grown dramatically in recent decades, and the disease is now endemic in more than 100 countries in Africa, the Americas, the Eastern Mediterranean, Southeast Asia and the Western Pacific [6,7]. The global resurgence of dengue is thought to be due to failure to control the *Aedes *populations, uncontrolled urbanization, population growth, climate change, and increasing number of international travelers [8].

In a review of the literature, we have found only one report describing the high resolution computed tomography (HRCT) findings in DHF [4]. The purpose of this study was to describe the high-resolution CT findings in a patient with dengue hemorrhagic fever, who presented with pulmonary hemorrhage and massive hemoptysis.

## Case presentation

**Figure 1:**
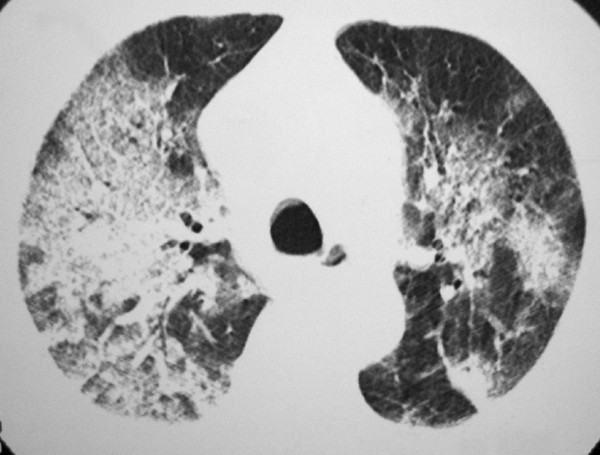
**HRCT showing extensive areas of consolidation with air bronchogram and ground glass opacities in both lungs, predominantly in the right lung**.

**Figure 2:**
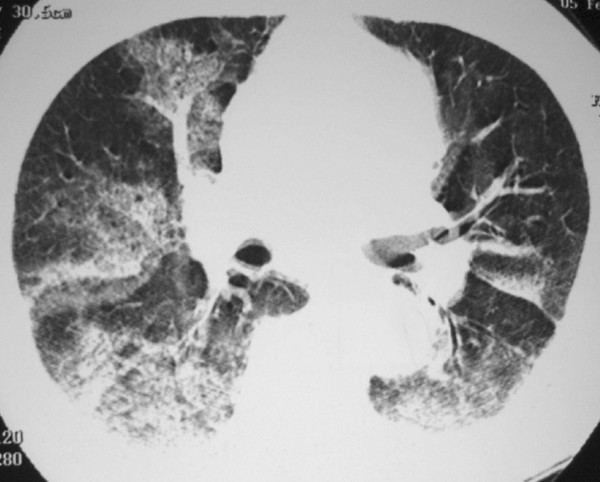
**HRCT showing extensive areas of consolidation with air bronchogram and ground glass opacities in both lungs, predominantly in the right lung**.

A 70-year-old male presented to the Emergency Department with massive hemoptysis for the last 6 hours, respiratory distress, anemia, hemodynamic instability, and renal failure. He had a 6-day history of fever, intense headache, retro-orbital pain, arthralgia, myalgias, and dry cough. There were no gastrointestinal complaints. Supportive measures were taken and the patient was admitted to the intensive care unit (ICU) and placed on mechanical ventilatory support. On examination, he appeared anxious, pale and dyspneic, and inspection revealed ecchymoses over his arms and abdomen. His vital signs included a tachycardia of 110 beats/min (bpm), a respiratory rate of 32 breaths/min, a blood pressure of 90/50 mmHg, and normal temperature (36.8°C). Fine bibasilar crackles were present bilaterally. The remainder of the physical examination was unremarkable. Laboratory evaluation revealed a red blood cell (RBC) count of 2.53 × 10^6^/mm^3^, hemoglobin level of 8.1 g/dL, hematocrit of 23.5% and platelet count of 50 × 10^3^/mm^3^. His WBC count was 4.1 × 10^3^/mm^3^, with a normal differential. Serum creatinine was 2.4 mg/dL, urea, 90 mg/dL, sodium, 135 mEq/L, aspartate aminotransferase, 30 U/L, alanine aminotransferase, 40 U/L, alkaline phosphatase, 200 U/L, gamma-glutamyl transpeptidase, 50 U/L, albumin, 2.5 g/dL, and globulin, 2.4 g/dL. Prothrombin time (PT) was 16.6s (65%) and international normalized ratio (INR) was 1.4. Arterial gasometry showed metabolic acidosis. Chest radiograph demonstrated bilateral condensations, and HRCT revealed large areas of consolidation and ground glass attenuation in both lungs, predominantly in the right lung, and small bilateral pleural effusions (Figure 1 and Figure 2). The patient then underwent bronchofibroscopy, which showed the presence of blood tinged secretion coming from both lungs, and friable mucosa with bleeding on touch. Lung biopsy was not performed because of coagulation abnormalities. Dengue serology, determined using a dengue IgM capture ELISA assay (MAC-ELISA), was positive. The patient required several blood transfusions, but developed refractory shock, severe metabolic acidosis, and died 15 days after admission.

## Discussion

The clinical presentations of dengue include non-specific febrile illness, dengue fever, dengue hemorrhagic fever, and dengue shock syndrome. Dengue fever usually presents as an acute fever with headache, rash, myalgia, retro-orbital pain, arthralgia, prostration, lymphadenopathy, and leucopenia [8,9]. In general, laboratorial findings include neutropenia followed by lymphocytosis, presence of atypical lymphocytes and thrombocytopenia [9]. Virus can be detected in blood for 4 or 5 days after the onset of symptoms and then disappears as antibody (immunoglobulin M [IgM]) production increases. In primary infections, IgG antibody appears within a few days, whereas in secondary infections, the IgG level rises immediately after the onset of symptoms and remains high in most patients [8].

Early diagnosis of DV infection is important and can be provisionally established by clinical observation and easily available laboratory tests. The presence of high fever of acute onset associated with a positive tourniquet test and hemoconcentration (increase of the hematocrit of 20% or more) or thrombocytopenia are sufficient to establish a provisional diagnosis of DHF, but a negative tourniquet test does not rule out dengue infection. Low platelet counts do not predict clinically significant bleeding in dengue, and DHF or dengue shock syndrome cases frequently have compensated consumptive coagulopathy that seldom requires treatment [7]. Therefore, platelet or blood transfusions are only indicated in cases where coagulopathy causes massive bleeding [10]. Currently, dengue diagnosis is based on serology, viral isolation and RNA detection. Enzyme-linked immunosorbent assays (ELISA) are still the most widely used technique for serological diagnosis, but they do not identify the dengue virus serotype responsible for the current infection [11].

According to WHO guidelines [10], our patient showed the criteria for defining a case of DHF, since he presented all of the following signs and symptoms: fever lasting 2–7 days; hemorrhagic tendencies, evidenced by the presence of ecchymoses and bleeding from the respiratory tract (hemoptysis); thrombocytopenia less than 100,000 cells per mm^3^; and evidences of plasma leakage, manifested by pleural effusion and hypoproteinaemia. Other manifestations of dengue fever were also present, including headache, myalgia, arthralgia, leucopenia and a positive IgM antibody test.

Dengue hemorrhagic fever is characterized by an increase in capillary permeability, which results in fluid extravasations (pleural effusion, ascites), and haemostatic changes, including decreased platelet levels near the time of defervescence and hemorrhagic manifestations [2,9]. Major hemorrhage is unusual except when in association with profound or prolonged shock [6]. The liver may be enlarged and serum activity of aminotransferases is usually increased [12]. DHF can also progress to DSS, which is associated with hypotension caused by severe plasma leakage [2,12].

Thoracic manifestations such as pleural effusion and pneumonitis, are uncommon in DHF, and pulmonary hemorrhage is even more rare. Hemoptysis has been reported in 1.4% of dengue infections. The pathogenesis of bleeding in DHF patients is not well understood. It is thought to be a multifactorial process with abnormalities in the coagulation cascade, thrombocytopenia, platelet dysfunction, disseminated intravascular coagulation, and vascular defects. Vascular permeability has been thought to be mediated by histamine release [4,5]. Our patient presented with hemoptysis, and, for this reason, he underwent HRCT for the investigation of pulmonary hemorrhage. HRCT revealed extensive areas of consolidation with air bronchogram and ground glass opacities in both lungs, predominantly in the right lung, and small bilateral pleural effusions. By contrast, the only report of the HRCT findings found in the literature described the presence of discrete patchy ground glass opacities in both lungs, which regressed after conservative treatment.

Morphological studies of lung tissues revealed interstitial pneumonia associated with focal or diffuse zones of alveolar congestion and hemorrhage, increase of alveolar macrophages number, recruiting of platelets, mononuclear, and polymorphonuclear cells [3,12].

There is no specific treatment for dengue fever. However, careful clinical management frequently saves the lives of DHF patients. With appropriate intensive supportive therapy, mortality may be reduced to less than 1 per cent [7].

## Conclusion

In conclusion, dengue hemorrhagic fever should be considered in the differential diagnosis of a patient with fever, hemoptysis, and diffuse pulmonary infiltration. The high-resolution CT findings of dengue hemorrhagic fever consist of bilateral areas of consolidation with air bronchogram and ground-glass opacities, and bilateral pleural effusions.

## Competing interests

The authors declare that they have no competing interests.

## Authors' contributions

EM conceived the study. GZ, CMM and ADV research the literature review and prepared the manuscript. CNB, JLNF and CAAN diagnosed the patient and drafted the initial manuscript. CNB and AASMDS wrote the presentation of the case, and contributed to the discussion section. EM and ADV edit and coordinated the manuscript. All authors read and approved the final manuscript.

## Consent

Written informed consent was obtained from the patient for publication of this case report and accompanying images. A copy of the written consent is available for review by the Editor-in-Chief of this journal.
